# Zone-specific acromial cortical thickening after reverse shoulder arthroplasty

**DOI:** 10.1007/s00264-026-06892-2

**Published:** 2026-07-02

**Authors:** Kazuhiro Ikeda, Shotaro Teruya, Hiromitsu Tsuge, Takeshi Makihara, Shinzo Onishi

**Affiliations:** 1 Department of Orthopaedic Surgery, Institute of Medicine, University of Tsukuba, Tsukuba, Japan; 2 Department of Orthopaedic Surgery, Kikkoman General Hospital, Noda, Japan; 3 Department of Orthopaedic Surgery, National Hospital Organization Kasumigaura Medical Center, Tsuchiura, Japan

**Keywords:** Reverse shoulder arthroplasty, Acromial fracture, Scapular spine fracture, Cortical thickening, Computed tomography, Stress fracture

## Abstract

**Purpose:**

Acromial and scapular spine fractures after reverse shoulder arthroplasty (RSA) can impair post-operative outcomes; however, quantitative assessment of pre-fracture cortical changes remains challenging. This study evaluated longitudinal changes in inferior acromial cortical thickness after RSA using a computed tomography-based (CT-based) acromial coronal view.

**Methods:**

This multicentre retrospective cohort study included 44 patients who underwent RSA. The acromion was divided into three zones: I (lateral acromion), II (acromial base), and III (scapular spine). Inferior cortical thickness was measured at the centre of each zone. Changes were analysed from immediately post-operatively to one year and from one to two years in shoulders with corresponding CT scans. Cortical area was assessed as a secondary outcome, and the fracture cases were described separately.

**Results:**

From immediately post-operatively to one year (*n* = 41), cortical thickness was unchanged in Zone I but increased in Zone II from 2.0 ± 0.5 to 2.4 ± 0.7 mm and in Zone III from 3.8 ± 1.0 to 4.6 ± 1.6 mm. From one to two years (*n* = 28), Zones II and III showed further increases, whereas Zone I remained unchanged. Thickness changes correlated with cortical area changes (Spearman’s ρ = 0.55). Five shoulders (11.4%) developed fractures within two years, and delayed Zone II and III fractures showed marked preceding cortical thickening.

**Conclusion:**

Inferior acromial cortical thickening after RSA progressed selectively in Zones II and III, suggesting region-specific post-operative acromial remodelling. These findings may help clarify the differences in fracture pathomechanisms according to the location.

**Supplementary Information:**

The online version contains supplementary material available at https://doi.org/10.1007/s00264-026-06892-2.

## Introduction

Reverse shoulder arthroplasty (RSA) is a widely used reconstructive procedure for shoulder disorders associated with rotator cuff dysfunction [1–3]. The semi-constrained implant design improves glenohumeral stability; medialisation of the centre of rotation and distalisation and lateralisation of the humerus enhance the mechanical advantage of the deltoid muscle [4]. Through this biomechanical reconstruction, RSA enables the restoration of active shoulder elevation even in patients with severe rotator cuff deficiency [1, 4]. However, these biomechanical changes may also increase stress around the acromion and scapular spine, leading to osseous complications including stress reactions and fractures [5]. Acromial and scapular spine fractures are among the most clinically important complications, occurring in approximately 2.8%–5.0% of patients after RSA [6–8] and potentially compromising post-operative shoulder elevation [9]. These fractures are increasingly understood not as simple traumatic fractures but as multifactorial stress-related fractures involving both bone fragility and RSA-specific post-operative mechanical loading. Consistent with this view, osteoporosis and low bone mineral density have been identified as patient-related risk factors [10–12], whereas glenoid lateralization, humeral distalization, and deltoid lengthening have been implicated as mechanical factors that may increase deltoid tension [11, 13–17]. Further supporting this stress-related pathophysiology, acromial stress reactions without a clearly visible fracture line have been described [5, 18]. These findings suggest that localised osseous changes may precede radiographically apparent fractures in this region. Therefore, detecting such pre-fracture changes may provide a basis for risk stratification and preventive strategies.

However, no standardised imaging method has been established to quantitatively evaluate pre-fracture osseous changes in the acromion and scapular spine after RSA. This limitation partly reflects the complex three-dimensional morphology of these structures, which makes it difficult to evaluate cortical changes over time on reproducible imaging planes [19]. Consequently, evidence regarding the location of acromial cortical changes after RSA and their progression over time remains limited, and these changes have not yet been established as imaging markers of fracture risk.

Therefore, this study aimed to quantify the longitudinal changes in acromial cortical thickness after RSA using a newly defined CT-based acromial coronal view. Specifically, we sought to characterise the zone-specific patterns of cortical thickening over time.

## Materials and methods

### Study design and ethics

This multicentre retrospective cohort study was conducted in accordance with the Declaration of Helsinki and approved by the Institutional Review Board of the primary institution (Approval No. R07-290; February 26, 2026). The affiliated institution also authorised the local study implementation. The requirement for written informed consent was waived due to the retrospective observational design, and an opt-out opportunity was provided through the public disclosure of study information.

The primary outcome was the inferior acromial cortical thickness measured on CT images obtained within one week after surgery and at one and two years post-operatively. Patients who developed acromial or scapular spine fractures during follow-up were excluded from the longitudinal cortical thickness analysis because post-fracture morphology could affect the measurements.

### Patients and baseline characteristics

**Fig. 1:**
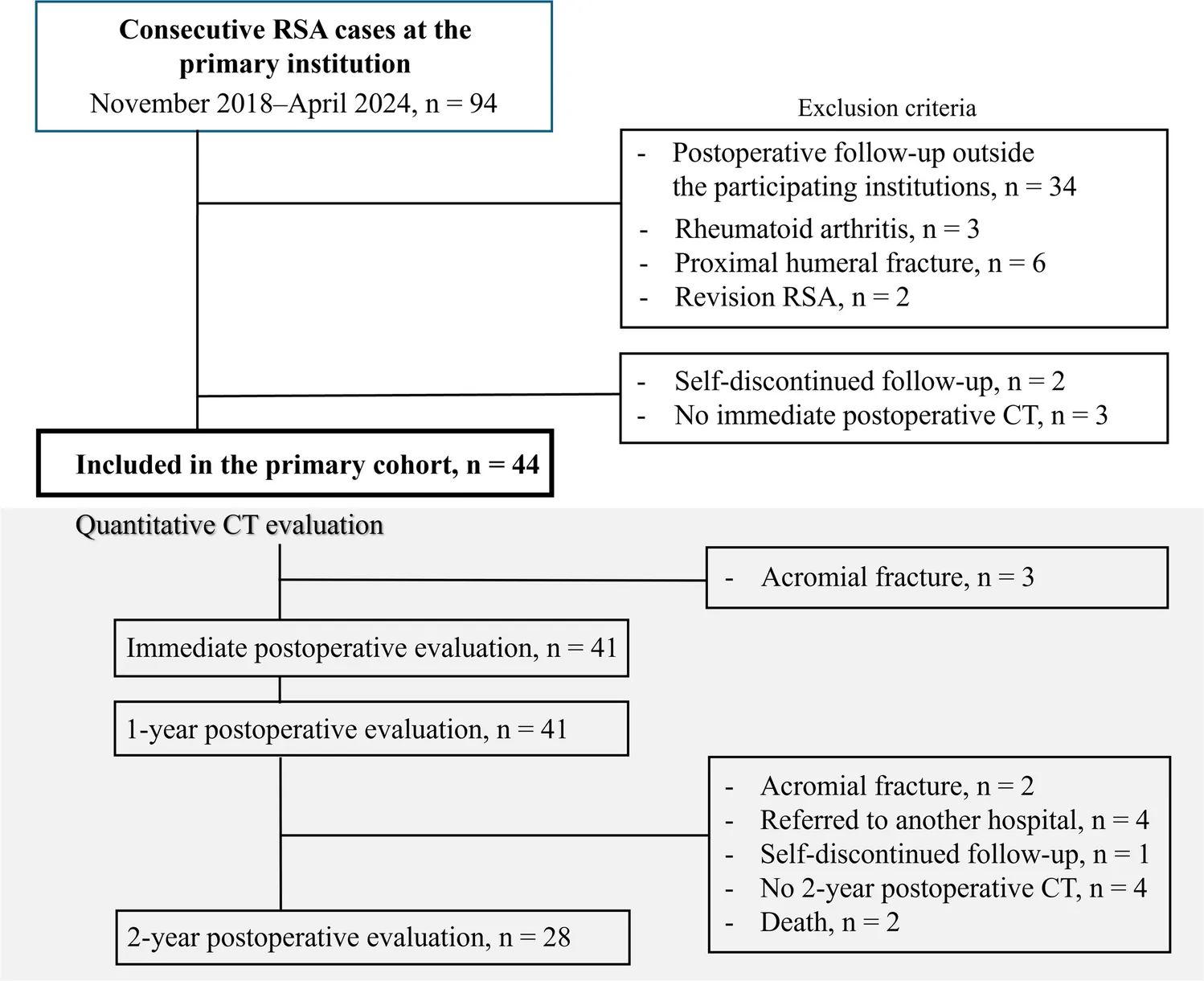
Flow diagram of patient selection and quantitative CT evaluation. Forty-four of 94 screened RSA cases were included in the primary cohort. Quantitative CT evaluation included 41 patients at the immediate post-operative and 1-year time points and 28 patients at the 2-year time point

Patients who underwent RSA at a primary institution between November 2018 and April 2024 were screened for eligibility (Fig. 1). Patients were eligible if CT images were available within one week after surgery and at one year post-operatively. To ensure consistency in CT acquisition, patients followed up at the referring hospitals were generally excluded, except for those followed up at affiliated institution B, where the same CT scanner model and imaging protocol were used. The exclusion criteria were rheumatoid arthritis, proximal humeral fracture, and revision RSA.

Of 94 screened RSAs, 44 were included in the final cohort study. Fracture cases were excluded from the longitudinal analyses after fracture occurrence; therefore, 41 shoulders were analysed immediately post-operatively to one year, and 28 shoulders were analysed from one to two years.

Demographic and clinical variables included age, sex, height, weight, body mass index (BMI), affected side, primary diagnosis, and Walch classification [20]. Operative variables included the use of bony increased-offset reverse shoulder arthroplasty (BIO-RSA) [21] and subscapularis repair. Radiographic variables included the lateralisation shoulder angle (LSA) and distalization shoulder angle (DSA), which were measured on immediate post-operative plain radiographs [22].

### Surgical procedure

The indications for RSA were irreparable massive rotator cuff tears, cuff tear arthropathy of Hamada grade 3 or higher [23], and primary osteoarthritis with Walch type B2, B3, or C glenoid deformity.

A single shoulder surgeon performed all procedures using a deltopectoral approach. In shoulders with an intact subscapularis tendon, subscapularis tenotomy was performed, and the tendon was repaired at wound closure. The coracoacromial ligament was released in all cases. In patients with Walch type A2 or higher glenoid bone loss, the bony increased-offset reverse shoulder arthroplasty technique described by Boileau et al. was used [24]. The Ascend Flex Reverse Shoulder System (Stryker, Kalamazoo, MI, USA) was used in all cases. The glenoid component was placed at a target RSA angle of 0° [25], and the humeral stem was inserted at 20° of retroversion.

The soft tissue tension was adjusted intraoperatively to achieve a stable reduction without overtensioning. Adequate tension was confirmed by smooth adduction of the arm to the trunk and the ability of the operated hand to reach the contralateral shoulder. Post-operatively, the patients used an abduction pillow brace for two weeks, after which range-of-motion exercises were allowed without restriction.

### CT acquisition

CT scans were obtained within one week after surgery, and one and two years post-opertively. Patients were scanned in the supine position with both arms placed alongside the trunk and palms against the lateral thighs. Axial volume data were acquired with a slice thickness of 1.0 mm, and single-energy metal artefact reduction (SEMAR) processing was applied to reduce metal artefacts. Detailed CT acquisition parameters are provided in Supplementary Table 1.

### CT reconstruction and quantitative assessment

**Fig. 2:**
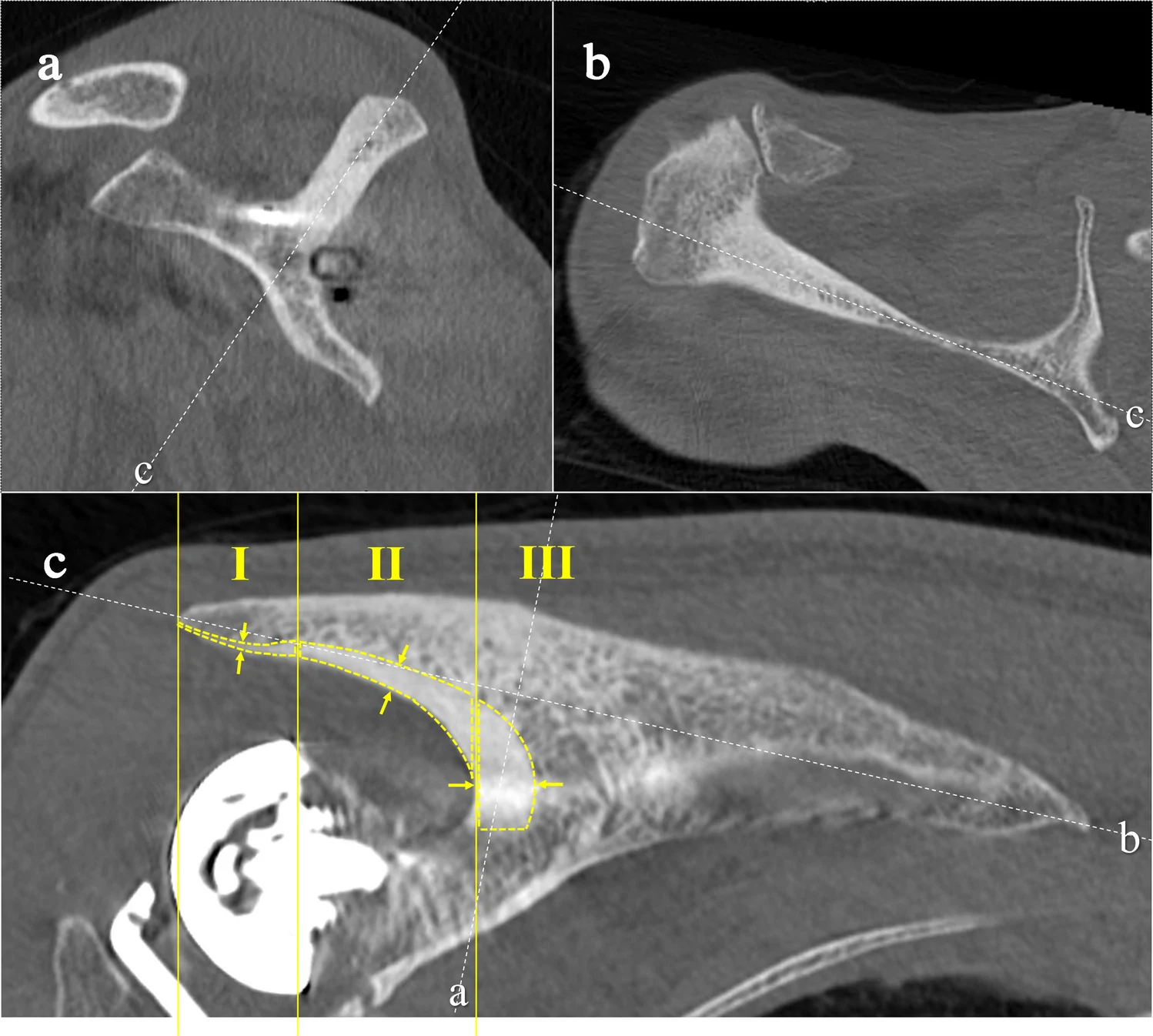
CT reconstruction, zoning, and measurement methods on the acromial coronal view. **a**, **b** Sagittal and axial reference planes. Dashed lines indicate the planes used to create the acromial coronal view by multiplanar reconstruction. **c** Reconstructed acromial coronal view. The acromion was divided into three zones: Zone I, lateral to the centre of rotation; Zone II, between the centre of rotation and the acromial base; and Zone III, medial to the acromial base. The centre of rotation was defined as the centre of the glenosphere. Inferior acromial cortical thickness was measured perpendicular to the cortical surface at the centre of each zone (arrows). Inferior cortical area was measured by tracing the inferior cortical region in each zone as a region of interest (yellow outline)

Multiplanar reconstruction (MPR) was performed using the acquired CT volume data to create a standardised CT-based acromial coronal view (Fig. 2). This view is defined as the coronal plane passing through the acromial base after adjusting the axial plane to continuously visualise the scapular spine.

In this view, the acromion is divided into three mediolateral zones according to the anatomical concept of the Levy classification [8]: Zone I, lateral to the centre of rotation; Zone II, between the centre of rotation and the acromial base; and Zone III, medial to the acromial base. The centre of rotation was defined as the centre of the glenosphere, and zoning reference lines were drawn parallel to the glenosphere inclination.

The primary outcome was the inferior acromial cortical thickness at the centre of each zone. The thickness was measured perpendicular to the inferior cortical surface. The inferior acromial cortical area was assessed as the secondary outcome by tracing the inferior cortical region in each zone as a region of interest.

All measurements were performed using RadiAnt DICOM Viewer (version 2025.2; Medixant, Poznań, Poland). Cortical thickness was measured independently by two shoulder surgeons (observer A, 13 years of experience; observer B, 10 years of experience). Each image was measured twice by each observer at least two weeks apart, and the mean of the two measurements by observer A was used for the primary analysis. Images were anonymised, randomised, and blinded to patient identifiers and imaging time points.

### Statistical analysis

Continuous variables are presented as means ± standard deviations. The Shapiro–Wilk test was used to assess normality. As the cortical thickness and area were not normally distributed, paired changes from immediately post-operatively to one year and from one to two years were analysed using the Wilcoxon signed-rank test for each zone. Bonferroni correction was applied for the two time-interval comparisons, with statistical significance set at *p* < 0.025.

Given its retrospective observational design, the sample size was based on all eligible patients during the study period. Accordingly, the effect size r was calculated to indicate the magnitude of change in cortical thickness and cortical area. The association between changes in cortical thickness and cortical area was evaluated using the Spearman’s rank correlation coefficient. Intraobserver and interobserver reliabilities for cortical thickness measurements were assessed using intraclass correlation coefficients (ICCs), with values interpreted as poor (≤ 0.40), fair (0.41–0.60), good (0.61–0.80), or excellent (0.81–1.00) [26, 27]. Statistical analyses were performed using Excel Statistics version 8.0 for Windows (Social Survey Research Information Co. Ltd., Tokyo, Japan).

## Results

The baseline patient and surgical characteristics are summarised in Table 1. The cohort had a mean age of 74.8 ± 6.1 years, and 32 patients (72.7%) were women. Massive rotator cuff tears or cuff tear arthropathy were the most common diagnoses (38 shoulders, 86.4%), and BIO-RSA was performed on 19 shoulders (43.2%). The post-operative lateralisation and distalization shoulder angles were 80.9 ± 12.1° and 57.2 ± 12.3°, respectively.

**Table 1:** Baseline patient and surgical characteristics

Category	Item	Results
Patient demographics	Sex, n	Men: 12; Women: 32
	Age	74.8 ± 6.1 years
	Height	151 ± 10 cm
	Body weight	59 ± 18 kg
	BMI	25.8 ± 7.8 kg/m^2^
	Dominant side affected, n (%)	23 (52.3%)
	Primary diagnosis, n (%)	Massive RCT or CTA: 38 (86.4%) Primary OA: 6 (13.7%)
	Walch classification, n (%)	A1: 23; A2: 12 B1: 0; B2: 2; B3: 2 C: 3; D: 2
Surgical variables	Subscapularis repair, n (%)	37 (84.1%)
	BIO-RSA technique, n (%)	19 (43.2%)
	Lateralization shoulder angle	80.9 ± 12.1°
	Distalization shoulder angle	57.2 ± 12.3°

Values are presented as mean ± standard deviation or number (%). *BMI* body mass index, *RCT* rotator cuff tear, *CTA* cuff tear arthropathy, *OA* osteoarthritis, *BIO-RSA* bony increased offset reverse shoulder arthroplasty

### Post-operative changes in cortical thickness and cortical area

**Fig. 3:**
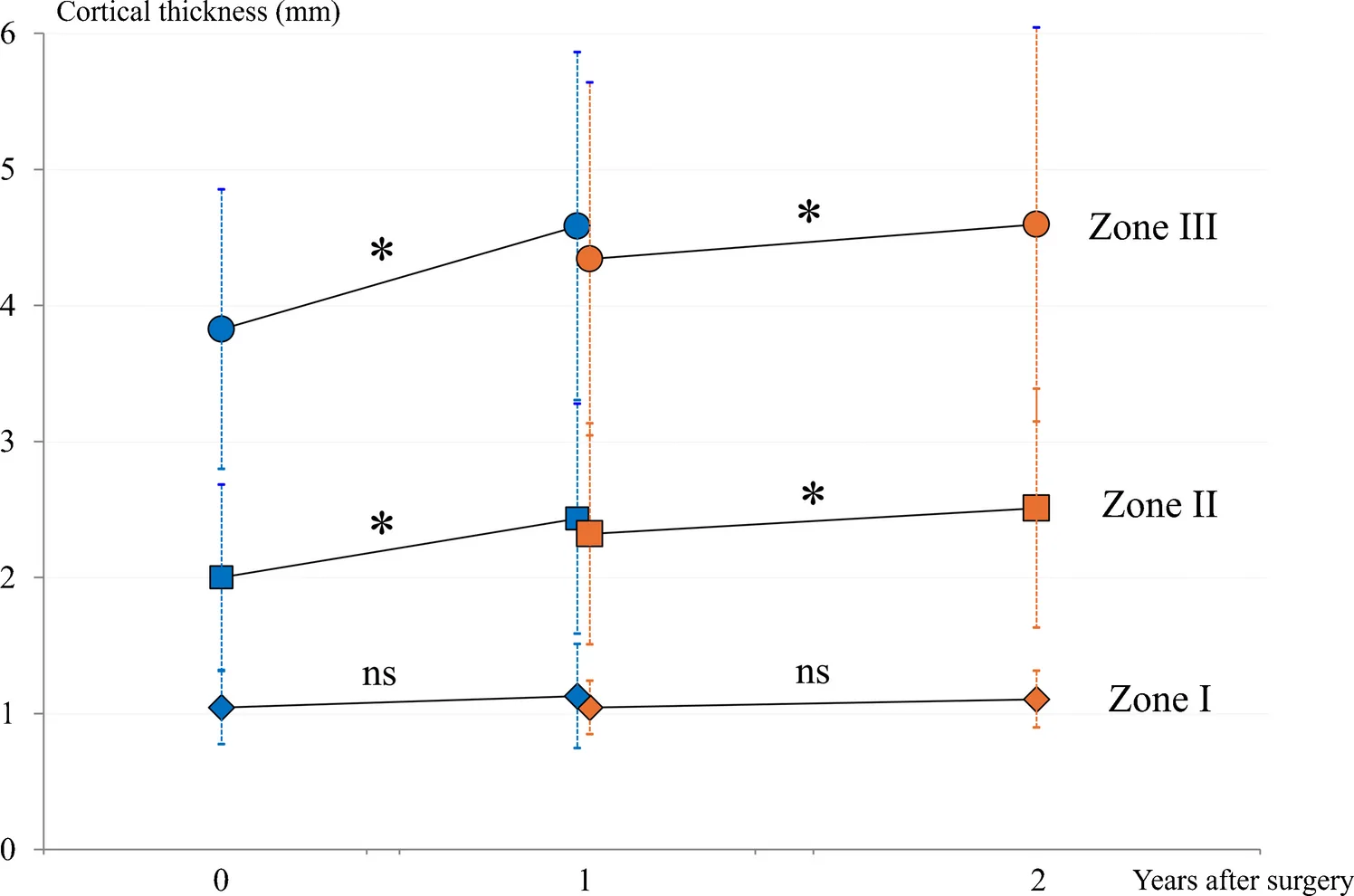
Temporal changes in inferior acromial cortical thickness after RSA. Inferior acromial cortical thickness is shown for each zone immediately post-operatively and at 1 and 2 years post-operatively. Diamonds, squares, and circles represent Zones I, II, and III, respectively. The blue markers and lines show changes from immediately to 1 year post-operatively (*n* = 41), whereas the orange markers and lines show changes from 1 to 2 years post-operatively (*n* = 28). Error bars indicate standard deviation. *p < 0.025

Temporal changes in cortical thickness and area are shown in Fig. 3 and Table 2, respectively. Cortical thickness showed zone-specific changes: Zone I showed no significant change during either interval, whereas Zones II and III increased significantly from immediately post-operatively to one year and from one to two years post-operatively, respectively. Cortical area showed a similar pattern, with significant increases in Zones II and III but not in Zone I. Changes in cortical thickness were moderately correlated with changes in cortical area (Spearman’s ρ = 0.55; *p* < 0.001).

**Table 2:** Temporal changes in inferior acromial cortical thickness and cortical area

Zone	Interval	Outcome	Baseline	Follow-up	P value	Effect size r
I	Immediate to 1 year	Thickness (mm)	1.0 ± 0.1	1.1 ± 0.1	0.042	0.36
		Area (mm^2^)	18.3 ± 9.6	20.5 ± 12.9	0.320	0.34
	1 to 2 years	Thickness	1.0 ± 0.2	1.1 ± 0.2	0.182	0.26
		Area	19.0 ± 12.9	20.3 ± 12.9	0.466	0.14
II	Immediate to 1 year	Thickness	2.0 ± 0.5	2.4 ± 0.7	< 0.001	0.54
		Area	52.1 ± 12.7	60.0 ± 16.7	< 0.001	0.54
	1 to 2 years	Thickness	2.3 ± 0.8	2.5 ± 0.9	0.021	0.46
		Area	54.9 ± 14.3	61.1 ± 22.1	0.017	0.45
III	Immediate to 1 year	Thickness	3.8 ± 1.0	4.6 ± 1.6	< 0.001	0.79
		Area	48.1 ± 19.0	64.2 ± 27.2	< 0.001	0.82
	1 to 2 years	Thickness	4.3 ± 1.6	4.5 ± 1.5	0.007	0.51
		Area	60.4 ± 28.1	66.4 ± 30.7	0.011	0.48

Values are presented as mean ± standard deviation. Comparisons were performed separately for two paired intervals: immediately to one year post-operatively and one to two years post-operatively. *P*-values were calculated using the Wilcoxon signed-rank test, with significance set at *p* < 0.025, after Bonferroni correction

The reliability of the cortical thickness measurements was excellent. The intraobserver ICC was 0.953 (95% CI, 0.942–0.963; *p* < 0.001), and the interobserver ICC was 0.913 (95% CI, 0.879–0.930; *p* < 0.001).

**Fig. 4:**
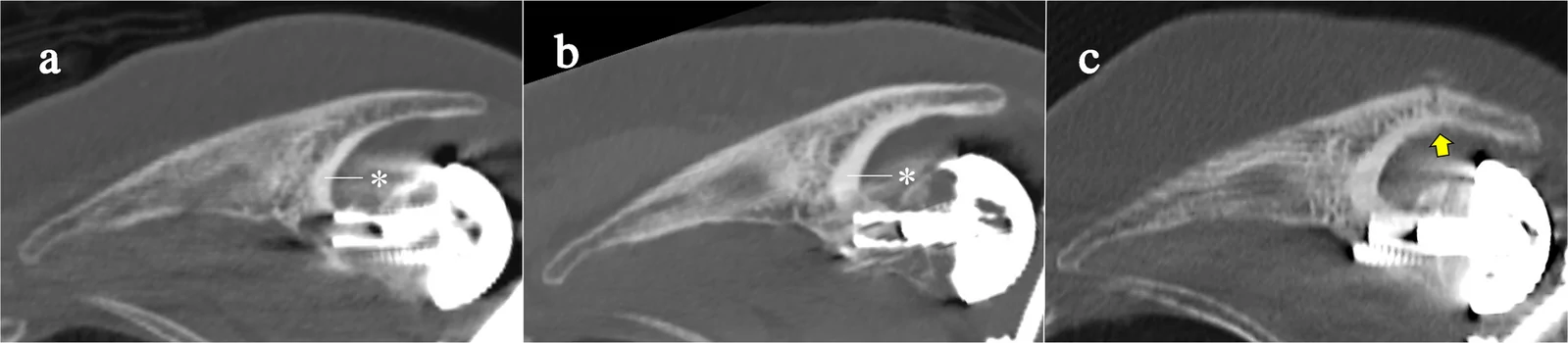
Representative case of delayed Zone II acromial fracture preceded by cortical thickening. CT-based acromial coronal views obtained immediately (**a**), 1 year (**b**), and 2 years post-operatively ^*^ (**c**) demonstrate progressive inferior cortical thickening in the corresponding zone before fracture detection (asterisks). Two years post-operatively, a fracture line was identified at the site of the preceding cortical thickening (yellow arrow). * The fracture-detection CT image was reconstructed with a 2-mm slice thickness and used for descriptive assessment only

Acromial or scapular spine fractures were observed in five patients (11.4%) within two years after RSA. Zone I fractures were observed in three patients; two were detected on the immediate post-operative CT and one was detected on the one year post-operative CT. Zone II and Zone III fractures were observed in one patient each, both of which were detected on the two year post-operative CT. In these two patients, cortical thickness in the corresponding zone had increased by 1.6 mm and 1.9 mm respectively, at one year post-surgery before fracture detection (Fig. 4). Both increases exceeded the 95th percentile of the cortical thickness changes in the overall cohort.

## Discussion

The principal finding of this study was that inferior acromial cortical thickening after RSA occurred selectively in Zones II and III, rather than uniformly across the acromion. This finding indicates that post-operative bone remodelling after RSA is localised regionally to the acromial base and scapular spine. Previous biomechanical studies have shown that stress and strain around the acromion and scapular spine after RSA are unevenly distributed and tend to concentrate from the acromial base toward the scapular spine [13, 15]. The distribution of cortical thickening observed in the present study was consistent with this biomechanical pattern.

This regional pattern suggests that acromial and scapular spine fractures after RSA may not represent a single uniform complication but rather site-dependent conditions with different pathophysiological mechanisms. If cortical thickening reflects an adaptive response to sustained mechanical loading, Zones II and III may be exposed to accumulated repetitive stress related to increased deltoid tension after RSA, which could contribute to delayed fatigue-type fractures. In the present series, delayed zones II and III fractures showed marked cortical thickening in the corresponding zone before fracture detection, which is consistent with this interpretation.

In contrast, Zone I fractures may involve different mechanisms. Previous studies have shown that acromial bone mineral density increases from lateral to medial, indicating that the lateral acromion is a relatively low-density region [28]. This anatomical characteristic may partly explain why Zone I fractures tend to occur relatively early and may be more closely related to bone fragility than to progressive stress accumulation. Consistent with this concept, the reported mean time to fracture was 6.6 ± 9.3 months for Levy type I fractures, 9.5 ± 11.9 months for Levy type II fractures, and 27.9 ± 35.1 months for Levy type III fractures [29], suggesting that lateral fractures may occur earlier than medial fractures. Therefore, risk assessment after RSA may need to distinguish between bone fragility in the lateral acromion and progressive cortical thickening around the acromial base and the scapular spine.

The standardised CT-based acromial coronal view enabled reproducible zone-specific quantification of cortical changes after RSA, as supported by excellent intra-and interobserver reliability. In addition, the moderate correlation between changes in cortical thickness and cortical area suggests that the inferior cortical thickness may reflect broader cortical remodelling. Thus, this measurement may provide a practical approach for evaluating post-operative acromial remodelling after RSA.

This study had several limitations. First, this was a retrospective observational study, and the cohort was limited to patients who underwent longitudinal CT evaluation at participating institutions, which may have introduced selection bias. Second, the implant design and surgical techniques were institution-specific. As glenoid lateralisation, inferior positioning of the glenoid component, and an onlay humeral construct may increase deltoid tension and acromial stress [11, 13–15, 30], these findings should be interpreted in the context of relatively high mechanical loading. Third, the number of fractures was limited. Therefore, we could not establish a direct relationship between cortical thickening and subsequent fractures; bone quality was also not evaluated, as DEXA, CT attenuation values, osteoporosis diagnoses, and osteoporosis-related medication data were unavailable. Larger longitudinal studies are needed to validate this association and determine clinically useful cutoff values.

## Conclusion

This study demonstrated that inferior acromial cortical thickening after RSA progressed selectively in Zones II (acromial base) and III (scapular spine), whereas Zone I (lateral acromion) showed no significant change up to two years post-operatively. These findings suggest region-specific post-operative acromial remodelling around the acromial base and scapular spine. Standardised CT-based assessments may help clarify the pathophysiology of post-RSA acromial lesions and support future fracture risk assessments.

## Supplementary Information

Below is the link to the electronic supplementary material.

ESM 1 (DOCX 14.7 KB)

## Data Availability

No datasets were generated or analysed during the current study.

## References

[1] Boileau P, Watkinson D, Hatzidakis AM, Hovorka I (2006) Neer Award 2005: the Grammont reverse shoulder prosthesis: results in cuff tear arthritis, fracture sequelae, and revision arthroplasty. J Shoulder Elbow Surg 15:527–540. 10.1016/j.jse.2006.01.003 16979046

[2] Buac NP, Chhokar M, Menendez ME (2026) Robotic-assisted reverse shoulder arthroplasty achieves operative time neutrality after an initial learning period. Int Orthop 50(4):853–857. 10.1007/s00264-026-06774-7 41820603

[3] Meissburger V, Housset V, Antoni M, Azar M, Mouchantaf M, Nourissat G (2026) No impact of osteoporosis on stemless reverse shoulder arthroplasty stability. Int Orthop 50(1):151–158. 10.1007/s00264-025-06683-1 41212189

[4] Werner CML, Steinmann PA, Gilbart M, Gerber C (2005) Treatment of painful pseudoparesis due to irreparable rotator cuff dysfunction with the Delta III reverse-ball-and-socket total shoulder prosthesis. J Bone Joint Surg Am 87:1476–1486. 10.2106/JBJS.D.02342 15995114

[5] Mayne IP, Bell SN, Wright W, Coghlan JA (2016) Acromial and scapular spine fractures after reverse total shoulder arthroplasty. Shoulder Elbow 8:90–100. 10.1177/1758573216628783 27583005 PMC4950466

[6] King JJ, Dalton SS, Gulotta LV, Wright TW, Schoch BS (2019) How common are acromial and scapular spine fractures after reverse shoulder arthroplasty? A systematic review. Bone Joint J 101–B:627–634. 10.1302/0301-620X.101B6.BJJ-2018-1187.R1 31154841

[7] Lau SC, Large R (2020) Acromial fracture after reverse total shoulder arthroplasty: a systematic review. Shoulder Elbow 12:375–389. 10.1177/1758573219876486 33281942 PMC7689606

[8] Levy JC, Anderson C, Samson A (2013) Classification of postoperative acromial fractures following reverse shoulder arthroplasty. J Bone Joint Surg Am 95:e104–e101. 10.2106/JBJS.K.01516 23925750

[9] Galvin JW, Eichinger JK, Li X, Parada SA (2022) Scapular fractures after reverse shoulder arthroplasty. J Am Acad Orthop Surg 30:e517–e527. 10.5435/JAAOS-D-20-01205 35050935

[10] Mahendraraj KA, Abboud J, Armstrong A et al (2021) Predictors of acromial and scapular stress fracture after reverse shoulder arthroplasty: a study by the ASES Complications of RSA Multicenter Research Group. J Shoulder Elbow Surg 30:2296–2305. 10.1016/j.jse.2021.02.008 33677115

[11] Cho CH, Rhee YG, Yoo JC et al (2021) Incidence and risk factors of acromial fracture following reverse total shoulder arthroplasty. J Shoulder Elbow Surg 30:57–64. 10.1016/j.jse.2020.04.031 32807375

[12] Colasanti CA, Lin CC, Levin JM et al (2025) Zone-specific bone density evaluation of the acromion may predict postoperative acromion stress fracture in patients undergoing a reverse total shoulder arthroplasty. J Shoulder Elbow Surg 34:e1005–e1016. 10.1016/j.jse.2025.02.013 40089016

[13] Kerrigan AM, Reeves J, Langohr GDG, Johnson JA, Athwal GS (2021) Reverse shoulder arthroplasty glenoid lateralization influences scapular spine strains. Shoulder Elbow 13:610–619. 10.1177/1758573220935567 34804210 PMC8600669

[14] Patterson BM, Johnson JE, Bozoghlian M, Anderson DD (2024) Increased deltoid and acromial stress with glenoid lateralization and onlay humeral stem constructs in reverse shoulder arthroplasty. J Shoulder Elbow Arthroplast. 10.1177/24715492241291311 PMC11497510 39444381

[15] Wong MT, Langohr GDG, Athwal GS, Johnson JA (2016) Implant positioning in reverse shoulder arthroplasty has an impact on acromial stresses. J Shoulder Elbow Surg 25:1889–1895. 10.1016/j.jse.2016.04.011 27374235

[16] Kriechling P, Weber F, Karczewski D, Borbas P, Wieser K (2023) Predictive factors of acromial fractures following reverse total shoulder arthroplasty: a subgroup analysis of 860 shoulders. JSES Int 7:812–818. 10.1016/j.jseint.2023.04.006 37719815 PMC10499654

[17] Lopiz Y, Rodrígiez-González A, Ossuna-Juntadez E, García-Fernández C, Martín-Albarrán S, Marco F (2026) Anterior deltoid atrophy after reverse shoulder arthroplasty: a preliminary prospective study on surgical approach and neurophysiological correlates. Int Orthop 50(5):1127–1134. 10.1007/s00264-026-06804-4 42024255 PMC13179907

[18] Kriechling P, Hodel S, Paszicsnyek A, Schwihla I, Borbas P, Wieser K (2022) Incidence, radiographic predictors, and clinical outcome of acromial stress reaction and acromial fractures in reverse total shoulder arthroplasty. J Shoulder Elbow Surg 31:1143–1153. 10.1016/j.jse.2021.11.012 34968697

[19] Sheth M, Khoo K, Telfer S, Schiffman C, Matsen F 3rd, Hsu J (2026) How much does radiographic projection affect the measurement of glenoid inclination? Int Orthop 50(3):619–624. 10.1007/s00264-026-06758-7 41711821

[20] Walch G, Badet R, Boulahia A, Khoury A (1999) Morphologic study of the glenoid in primary glenohumeral osteoarthritis. J Arthroplasty 14:756–760. 10.1016/s0883-5403(99)90232-2 10512449

[21] Boileau P, Moineau G, Roussanne Y, O’Shea K (2011) Bony increased-offset reversed shoulder arthroplasty: minimizing scapular impingement while maximizing glenoid fixation. Clin Orthop Relat Res 469:2558–2567. 10.1007/s11999-011-1775-4 21286887 PMC3148388

[22] Boutsiadis A, Lenoir H, Denard PJ et al (2018) The lateralization and distalization shoulder angles are important determinants of clinical outcomes in reverse shoulder arthroplasty. J Shoulder Elbow Surg 27:1226–1234. 10.1016/j.jse.2018.02.036 29602633

[23] Hamada K, Fukuda H, Mikasa M, Kobayashi Y (1990) Roentgenographic findings in massive rotator cuff tears. A long-term observation. Clin Orthop Relat Res 254:92–96. 10.1097/00003086-199005000-00014 2323152

[24] Boileau P, Moineau G, Roussanne Y, O’Shea K (2017) Bony increased offset-reversed shoulder arthroplasty (BIO-RSA). JBJS Essent Surg Tech 7:e37. 10.2106/JBJS.ST.17.00006 30233972 PMC6132994

[25] Boileau P, Gauci MO, Wagner ER et al (2019) The reverse shoulder arthroplasty angle: a new measurement of glenoid inclination for reverse shoulder arthroplasty. J Shoulder Elbow Surg 28:1281–1290. 10.1016/j.jse.2018.11.074 30935825

[26] Ikeda K, Ogawa T, Ikumi A et al (2024) Magnetic resonance imaging predicts outcomes of conservative treatment in patients with lateral epicondylitis. J Orthop Sci 29:795–801. 10.1016/j.jos.2023.03.014 37024365

[27] Ikeda K, Ogawa T, Ikumi A et al (2022) Individual evaluation of the common extensor tendon and lateral collateral ligament improves the severity diagnostic accuracy of magnetic resonance imaging for lateral epicondylitis. Diagnostics (Basel) 12:1871. 10.3390/diagnostics12081871 36010221 PMC9406652

[28] Voss A, Dyrna F, Achtnich A et al (2017) Acromion morphology and bone mineral density distribution suggest favorable fixation points for anatomic acromioclavicular reconstruction. Knee Surg Sports Traumatol Arthrosc 25:2004–2012. 10.1007/s00167-017-4539-1 28434036

[29] Marigi EM, Eboh S, Marigi IM et al (2025) Acromial and scapular fractures after reverse shoulder arthroplasty: comparison of 3018 reverse total shoulders by inlay and onlay humeral component design. J Shoulder Elbow Surg 34:1507–1513. 10.1016/j.jse.2024.09.029 39579858

[30] Cirello A, Ingrassia T, Rovere G, Nalbone L, Camarda L, Mirulla IA, Nigrelli V, Ricotta V, Tantillo M (2026) Customized positioning of the glenoid component in reverse shoulder arthroplasty: a new computer aided design methodology. Int Orthop 50(3):625–636. 10.1007/s00264-026-06748-9 41653229 PMC12992450

