# Supplementary material for: Zone-specific acromial cortical thickening after reverse shoulder arthroplasty
Source: Int Orthop. 2026 Jul 2;50(9):2377–84. doi: 10.1007/s00264-026-06892-2 (PMC13562106; doi:10.1007/s00264-026-06892-2)
Supplement: Supplementary file 1 [file 264_2026_6892_MOESM1_ESM.docx]

| Parameter | Value |
| --- | --- |
| CT manufacturer | Canon |
| CT scanner model | Aquilion ONE |
| Tube voltage | 135 kVp |
| Tube current | Automatic exposure control according to patient body size |
| Pitch | 0.825 |
| Rotation time | 0.75 s |
| Field of view | 240 mm |
| Slice thickness | 1.0 mm |
| Metal artifact reduction | Single-energy metal artifact reduction（SEMAR） |

Table S1. CT parameter
